# Synthesis of FeN_4_ at 180 GPa and its crystal structure from a submicron-sized grain

**DOI:** 10.1107/S2056989018012161

**Published:** 2018-09-07

**Authors:** Maxim Bykov, Saiana Khandarkhaeva, Timofey Fedotenko, Pavel Sedmak, Natalia Dubrovinskaia, Leonid Dubrovinsky

**Affiliations:** a Bayerisches Geoinstitut, University of Bayreuth, 95440 Bayreuth, Germany; b Material Physics and Technology at Extreme Conditions, Laboratory of Crystallography, University of Bayreuth, 95440 Bayreuth, Germany; c European Synchrotron Radiation Facility, BP 220, 38043 Grenoble Cedex, France

**Keywords:** polynitrides, iron tetra­nitride, high-pressure single-crystal X-ray diffraction, crystal structure

## Abstract

Iron tetra­nitride, FeN_4_, was synthesized from the elements in a laser-heated diamond anvil cell at 180 (5) GPa and 2700 (200) K. Its crystal structure was determined based on single-crystal X-ray diffraction data collected from a submicron-sized grain at the synchrotron beamline ID11 of ESRF. The compound crystallizes in the triclinic space group *P* . In the asymmetric unit, the Fe atom occupies an inversion centre (Wyckoff position 1*d*), while two N atoms occupy general positions (2*i*). The structure is made up from edge-sharing [FeN_6_] octa­hedra forming chains along [100] and being inter­connected through N—N bridges. N atoms form *catena*-poly[tetraz-1-ene-1,4-di­yl] anions [–N=N—N—N–]_∞_^2−^ running along [001]. In comparison with the previously reported structure of FeN_4_ at 135 GPa [Bykov *et al.* (2018). *Nat. Commun.* **9**, 2756], the crystal structure of FeN_4_ at 180 GPa is similar but the structural model is significantly improved in terms of the precision of the bond lengths and angles.

## Abstract 2

The refined crystal structure model of iron tetra­nitride, FeN_4_, at 180 GPa is similar to that at 135 GPa but shows improved structural parameters in terms of bond lengths and angles.

## Chemical context   

Polynitro­gen compounds have attracted great inter­est because of their potential applications as high-energy-density materials. Recently, a variety of nitro­gen-rich compounds containing polymeric and oligomeric nitro­gen chains, N_5_ or N_6_ rings, or even more complex networks have been predicted to be stable at high pressures (Steele & Oleynik, 2016 ▸, 2017 ▸; Zhang *et al.*, 2017 ▸; Xia *et al.*, 2018 ▸). Predicted lithium and caesium penta­zolates LiN_5_ and CsN_5_ were successfully synthesized at high-pressure conditions *via* the reaction between a metal or metal azide and nitro­gen (Laniel *et al.*, 2018 ▸; Steele *et al.*, 2017 ▸). Recently, Bykov and co-workers synthesized two compounds containing polymeric nitro­gen chains, *viz*. an inclusion compound ReN_8_·N_2_ (Bykov *et al.*, 2018*b* ▸) and iron tetra­nitride, FeN_4_ (Bykov *et al.* 2018*a* ▸) *via* the reaction between Fe or Re and nitro­gen in a laser-heated diamond anvil cell (DAC). The crystal structures of these compounds were studied at pressures up to 135 GPa by means of single-crystal X-ray diffraction (SCXRD).

The higher the pressures, the more challenging are synthesis and diffraction studies in DACs, even at dedicated high-pressure stations at the 3rd generation synchrotron facilities where the X-ray beam can be focused down to 2–3 µm. First of all, at pressures exceeding 150 GPa, the size of the sample is of only about 10 µm or less, and single-crystalline grains of the reaction product(s) are often of submicron size, which results in a drastic worsening of the signal-to-noise ratio in SCXRD. Additionally, the contribution of parasitic diffraction from the gasket material increases with pressure because the sample chamber becomes smaller upon compression. Submicron focusing of the X-ray beam, which is possible on some synchrotron beamlines, can provide suitable conditions to collect SCXRD data at multi-megabar pressures. Here we report the synthesis of FeN_4_ from the elements at a pressure of about 180 GPa and provide the structure refinement for FeN_4_ against SCXRD data at this pressure. The X-ray beam focusing down to 0.3×0.3 µm^2^ at the synchrotron beamline ID11 (ESRF, Grenoble, France) allowed us to collect SCXRD data from an FeN_4_ grain with linear dimensions of about 0.5 µm.

## Structural commentary   

**Figure 1:**
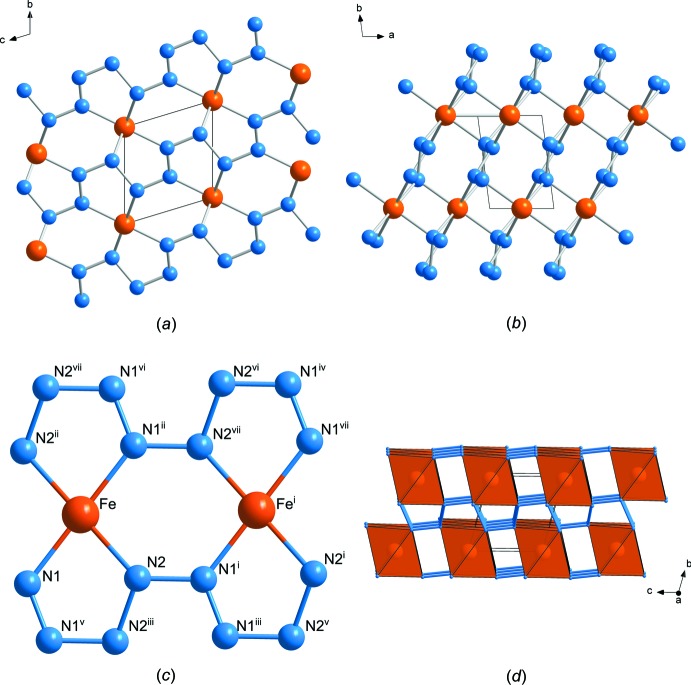
The crystal structure of FeN_4_ at 180 GPa. (*a*) and (*b*) Projections of the crystal structure along [100] and [001], respectively. (*c*) A fragment of the crystal structure showing the coordination of Fe atoms. [Symmetry codes: (i) *x*, *y*, 1 + *z*; (ii) 1 − *x*, −*y*, −*z*; (iii) −*x*, 1 − *y*, −*z*; (iv) 1 + *x*, 1 + *y*, 2 + *z*; (v) −*x*, −1 − *y*, −1 − *z*; (vi) 1 + *x*, 1 + *y*, 1 + *z*; (vii) 1 − *x*, −*y*, 1 − *z*; (viii) 1 + *x*, 1 + *y*, *z*.] (*d*) The crystal structure of FeN_4_ in polyhedral representation.

**Figure 2:**
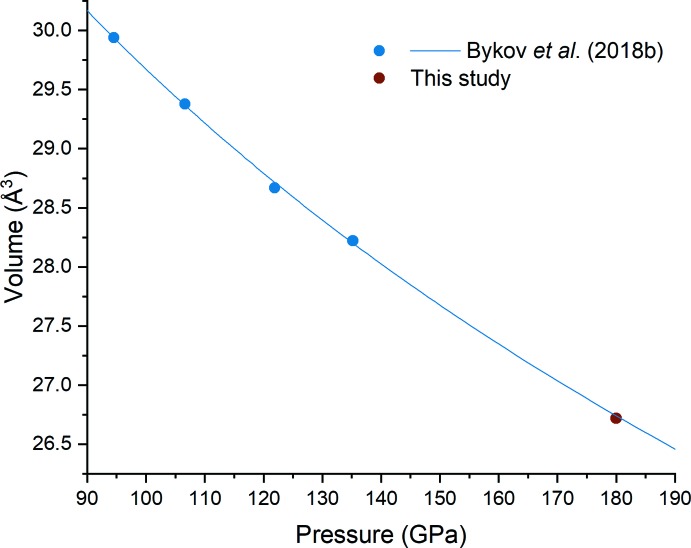
Pressure-dependence of the unit-cell volume of FeN_4_. Blue points and the equation of state (blue line) are taken without modification from Bykov *et al.* (2018*a* ▸) [*V*_94.5_ = 29.94 (4) Å^3^, *K*_94.5_ = 603 (22) GPa, *K*′_94.5_ = 4.0 (fixed)]. Red point – current study.

The crystal structure (Fig. 1 ▸ *a*,*b*) and the unit-cell volume (Fig. 2 ▸) of FeN_4_ at 180 GPa are in a good agreement with the structural model for this compound at 135 GPa and its equation of state as reported by Bykov *et al.* (2018*a* ▸). Despite the increased pressure, as a result of the application of the submicron beam focusing, the quality of the SCXRD data collected at 180 GPa turned out to be much better. Thus, the quality of the structure refinement of FeN_4_ based on the 180 GPa data set is significantly improved in comparison with that for the 135 GPa data set. This is evident from a comparison of such important refinement indicators such as the data-to-parameter ratio (7.1 *vs* 4.8), Δρ_max_/Δρ_min_ (0.76/−0.56 *vs* 0.98/−1.09 e Å^−3^) and *R*_1_[*I*>2σ(*I*)] (0.040 *vs* 0.064). Furthermore, the precision of the bond lengths and angles is significantly improved (Table 1 ▸).

The Fe1 atom occupies an inversion centre of space-group type *P* 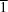 (Wyckoff position 1*d*), while the two nitro­gen atoms N1 and N2 occupy general positions (2*i*). The iron atom is coordinated by six nitro­gen atoms, forming a distorted octa­hedron. [FeN_6_] octa­hedra share opposite edges, thus forming infinite chains along [100]. These chains are inter­connected through N—N bridges as shown in Fig. 1 ▸ *d*. The covalently bonded nitro­gen atoms form infinite zigzag chains running along [001] (Fig. 1 ▸ *a*–*c*). The N1 atom has a trigonal–planar coordination, while N2 is tetra­hedrally coordinated, suggesting *sp* ^2^ and *sp* ^3^ hybridization, respectively. In agreement with the study of Bykov *et al.* (2018*a* ▸), the N—N distances increase in the following order *d*(N1—N1) < *d*(N1—N2) < *d*(N2—N2) (Table 1 ▸), supporting the conclusion that the N1—N1 bond is a double-bond, while N1—N2 and N2—N2 bonds are single bonds. Therefore, the nitro­gen atoms form *catena*-poly[tetraz-1-ene-1,4-di­yl] anions [–N=N—N—N–]_∞_^2–^.

The key parameters for the synthesis of polynitrides are pressure–temperature conditions and the choice of metal and/or nitro­gen precursors. High temperatures and pressures are required to overcome the kinetic barrier for breaking the triple N≡N bond, to increase the chemical potential of nitro­gen and to stabilize the reaction products (Sun *et al.*, 2017 ▸). It is known that increasing pressure allows compounds with higher nitro­gen content to be obtained, *e.g.* for the Fe—N system Fe_*x*_N (*x* = 2–8) can be synthesized at ambient and low pressures (Ertl *et al.* 1979 ▸), FeN at 12 GPa (Clark *et al.*, 2017 ▸), FeN_2_ at 60 GPa, and FeN_4_ at 106 GPa (Bykov *et al.*, 2018*a* ▸). Inter­estingly, at a given pressure, different metals stabilize different types of nitro­gen networks. For example, ReN_8_·N_2_ synthesized at 106 GPa contains polydiazene chains [–N=N–]_∞_ (Bykov *et al.*, 2018*b* ▸), whereas alkali metals form penta­zolate salts at even lower pressures (Laniel *et al.*, 2018 ▸; Steele *et al.*, 2017 ▸), *i.e*. the type of metal, the variety of its oxidation states, and its ionic radius play an important role in the chemistry of the nitro­gen network. The current study shows that FeN_4_ can be synthesized in a broad pressure range from 106 to 180 GPa. Such an extended stability range for this compound may be related to the favourable sixfold coordination of Fe. On one hand, it perfectly matches the 18 *e* ^−^ rule (Bykov *et al.*, 2018*a* ▸), and on the other hand, for the Fe—N system coordination number 6 is geometrically preferable. Further systematic studies of various metal polynitrides will allow empirical rules for the design of novel materials at different pressure and temperature conditions to be formulated.

## Synthesis and crystallization   

A piece of iron powder (Sigma Aldrich, 99.99%) was loaded inside a sample chamber of a BX90-type diamond anvil cell equipped with double-bevelled Boehler–Almax type diamonds (culet diameter 40 µm). Nitro­gen was used as a pressure-transmitting medium and as a reagent for the synthesis. The sample was compressed up to 180 GPa and laser-heated from both sides up to 2700 (200) K. The pressure was determined using the equation of state (EoS) of *hcp*-iron. As there are several equations of state of iron in the literature (Table 2 ▸), for a given unit-cell volume of iron *V*_Fe_ = 15.171 (5) Å^3^ one can get slightly different pressures in the range 173.5 to 187.5 GPa with an average of 179.8(5.2) GPa. Taking into account this uncertainty in the pressure determination, we accepted the rounded value of 180 GPa.

**Figure 3:**
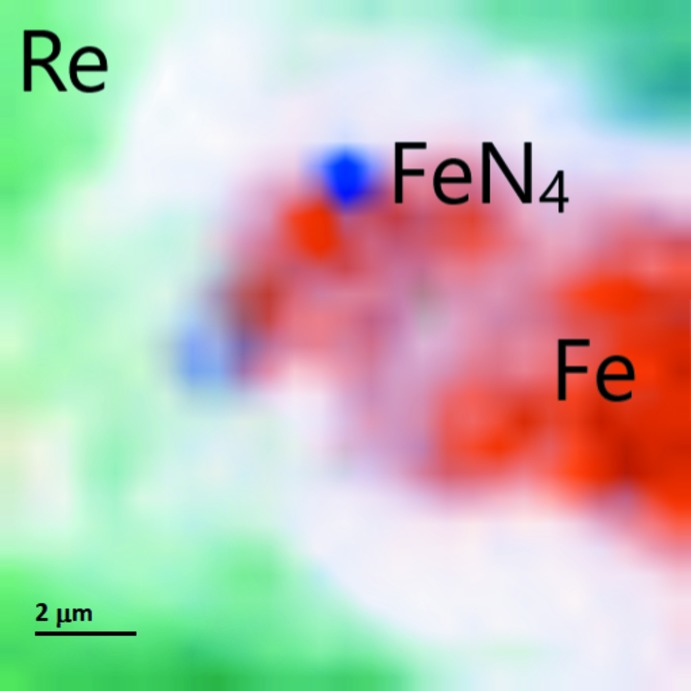
X-ray diffraction imaging of the sample chamber at 180 GPa. The colour intensity is proportional to the intensity of the following reflections: the (100) reflection of Re for the green region; the (101) reflection of Fe for the orange region; the sum of the (101), (1
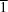
1), (0 0 2), and (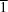 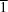
2) reflections of FeN_4_ for the blue region.

In order to locate the FeN_4_ grain in the sample chamber we used the following strategy: we collected 27 × 27 = 729 still images with the exposure time of 6 s. Before taking the next image, either the horizontal or vertical motor was moved by 0.5 µm, allowing a 13 × 13 µm^2^ X-ray diffraction map of the sample chamber to be built up (Fig. 3 ▸). The images were then analyzed with *XDI* software (Hrubiak, 2017 ▸).

## Refinement   

Crystal data, data collection details and structure refinement details are summarized in Table 3 ▸. We have used the same non-reduced unit-cell setting and the structure model of FeN_4_ at 135 GPa (Bykov *et al.*, 2018*a* ▸) for refinement of the crystal structure of FeN_4_ at 180 GPa. As a result of the limited angular range caused by the laser-heated DAC and the very small crystal size, the resolution of the data set was not sufficient to refine the atoms with anisotropic displacement parameters. Hence they were refined with isotropic displacement parameters only.

## Supplementary Material

Crystal structure: contains datablock(s) I. DOI: 10.1107/S2056989018012161/wm5459sup1.cif

Structure factors: contains datablock(s) I. DOI: 10.1107/S2056989018012161/wm5459Isup2.hkl

CCDC reference: 1864279

Additional supporting information:  crystallographic information; 3D view; checkCIF report

## Figures and Tables

**Table 1:** Selected bond lengths for FeN_4_ at 135 GPa (Bykov *et al.*, 2018*a* ▸) and at 180 GPa (this study)

	135 GPa	180 GPa
Fe—N1	1.73 (2)	1.707 (10)
Fe—N1^i^	1.73 (2)	1.707 (10)
Fe—N2^ii^	1.81 (3)	1.783 (14)
Fe—N2^iii^	1.81 (3)	1.783 (14)
Fe—N2^iv^	1.78 (3)	1.763 (6)
Fe—N2^v^	1.78 (3)	1.763 (6)
N1—N1^vi^	1.29 (5)	1.277 (14)
N1—N2	1.30 (3)	1.298 (8)
N2—N2^vii^	1.43 (4)	1.37 (3)

Symmetry codes: (i) −*x* + 1, −*y*, −*z*; (ii) *x* + 1, *y*, *z* + 1; (iii) −*x*, −*y*, −*z* − 1; (iv) −*x* + 1, −*y*, −*z* − 1; (v) *x*, *y*, *z* + 1; (vi) −*x*, −*y* − 1, −*z* − 1; (vii) −*x*, −*y* − 1, −*z* − 2.

**Table 2:** Pressures for FeN_4_ synthesis based on different 3rd order Birch–Murnaghan EoS’s of *hcp*-Fe reported in the literature [*V*_Fe_ = 15.171 (5) Å^3^/unit cell]

Reference	*V*_0_ (Å^3^)	*K* (GPa)	*K*′	Pressure (GPa)
Dewaele *et al.* (2006 ▸)	22.468 (24)	165 (fixed)	4.97 (4)	173.5(2.2)
Fei *et al.* (2016 ▸)	22.428 (fixed)	172.7(1.4)	4.79 (5)	174.1(1.4)
Sakai *et al.* (2014 ▸)	22.18 (20)	179.6(2.2)	4.91 (12)	174.9(2.1)
Mao *et al.* (1990 ▸)	22.35 (3)	164.8(3.6)	5.33 (9)	179.8(4.3)
Yamazaki *et al.* (2012 ▸)	22.15 (5)	202 (7)	4.5 (2)	181.0(5.6)
Dubrovinsky *et al.* (2000 ▸)	22.35 (3)	155.6(3.5)	5.81 (6)	183.7(4.8)
Garai *et al.* (2011 ▸)	22.33 (3)	164 (2)	5.52 (5)	183.9(2.5)
Boehler *et al.* (2008 ▸)	22.46 (4)	160 (6)	5.6 (2)	187.5(8.2)

**Table 3:** Experimental details

Crystal data	
Chemical formula	FeN_4_
*M*_r_	111.89
Crystal system, space group	Triclinic, *P* 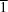
Temperature (K)	293
*a*, *b*, *c* (Å)	2.4473 (10), 3.4688 (14), 3.5144 (13)
α, β, γ (°)	105.22 (4), 110.60 (4), 91.39 (3)
*V* (Å^3^)	26.72 (2)
*Z*	1
Radiation type	Synchrotron, λ = 0.30996 Å
μ (mm^−1^)	1.33
Crystal size (mm)	0.0005 × 0.0005 × 0.0005

Data collection	
Diffractometer	ID11 @ ESRF
Absorption correction	Multi-scan (*ABSPACK*; Oxford Diffraction, 2005 ▸)
*T*_min_, *T*_max_	0.967, 1.000
No. of measured, independent and observed [*I* > 2σ(*I*)] reflections	117, 71, 70
*R*_int_	0.020
(sin θ/λ)_max_ (Å^−1^)	0.901

Refinement	
*R*[*F* ^2^ > 2σ(*F* ^2^)], *wR*(*F* ^2^), *S*	0.040, 0.082, 1.18
No. of reflections	71
No. of parameters	10
Δρ_max_, Δρ_min_ (e Å^−3^)	0.76, −0.56

Computer programs: *CrysAlis PRO* (Rigaku OD, 2018 ▸), *SHELXT* (Sheldrick, 2015*a* ▸), *SHELXL2014* (Sheldrick, 2015*b* ▸) and *OLEX2* (Dolomanov *et al.*, 2009 ▸).

## supplementary crystallographic information

### Crystal data

**Table:**

FeN_4_	*Z* = 1
*M**_r_* = 111.89	*F*(000) = 54
Triclinic, *P*1	*D*_x_ = 6.953 Mg m^−^^3^
*a* = 2.4473 (10) Å	Synchrotron radiation, λ = 0.30996 Å
*b* = 3.4688 (14) Å	Cell parameters from 68 reflections
*c* = 3.5144 (13) Å	θ = 2.8–16.1°
α = 105.22 (4)°	µ = 1.33 mm^−^^1^
β = 110.60 (4)°	*T* = 293 K
γ = 91.39 (3)°	Irregular, black
*V* = 26.72 (2) Å^3^	0.001 × 0.001 × 0.001 mm

### Data collection

**Table:**

ID11 @ ESRF diffractometer	71 independent reflections
Radiation source: synchrotron	70 reflections with *I* > 2σ(*I*)
Synchrotron monochromator	*R*_int_ = 0.020
ω scans	θ_max_ = 16.2°, θ_min_ = 2.8°
Absorption correction: multi-scan (*ABSPACK*; Oxford Diffraction, 2005)	*h* = −3→3
*T*_min_ = 0.967, *T*_max_ = 1.000	*k* = −5→4
117 measured reflections	*l* = −6→5

### Refinement

**Table:**

Refinement on *F*^2^	0 restraints
Least-squares matrix: full	Primary atom site location: dual
*R*[*F*^2^ > 2σ(*F*^2^)] = 0.040	*w* = 1/[σ^2^(*F*_o_^2^) + (0.0282*P*)^2^ + 0.3122*P*] where *P* = (*F*_o_^2^ + 2*F*_c_^2^)/3
*wR*(*F*^2^) = 0.082	(Δ/σ)_max_ < 0.001
*S* = 1.18	Δρ_max_ = 0.76 e Å^−^^3^
71 reflections	Δρ_min_ = −0.56 e Å^−^^3^
10 parameters	

### Special details

**Table:**

Geometry. All esds (except the esd in the dihedral angle between two l.s. planes) are estimated using the full covariance matrix. The cell esds are taken into account individually in the estimation of esds in distances, angles and torsion angles; correlations between esds in cell parameters are only used when they are defined by crystal symmetry. An approximate (isotropic) treatment of cell esds is used for estimating esds involving l.s. planes.

### Fractional atomic coordinates and isotropic or equivalent isotropic displacement parameters (Å^2^)

**Table:**

	*x*	*y*	*z*	*U*_iso_*/*U*_eq_	
Fe	0.5000	0.0000	0.0000	0.0072 (4)*	
N1	0.163 (4)	−0.346 (4)	−0.485 (2)	0.0066 (10)*	
N2	0.065 (3)	−0.309 (4)	−0.861 (2)	0.0068 (10)*	

### Geometric parameters (Å, º)

**Table:**

Fe—Fe^i^	2.4473 (10)	Fe—N2^vii^	1.763 (6)
Fe—Fe^ii^	2.4473 (10)	N1—N1^viii^	1.277 (14)
Fe—N1	1.707 (10)	N1—N2	1.298 (8)
Fe—N1^iii^	1.707 (10)	N2—Fe^ix^	1.763 (6)
Fe—N2^iv^	1.783 (14)	N2—Fe^x^	1.783 (14)
Fe—N2^v^	1.783 (14)	N2—N2^xi^	1.37 (3)
Fe—N2^vi^	1.763 (6)		
			
Fe^i^—Fe—Fe^ii^	180.0	N2^vii^—Fe—Fe^ii^	46.7 (5)
N1—Fe—Fe^i^	96.6 (4)	N2^iv^—Fe—Fe^ii^	133.98 (18)
N1^iii^—Fe—Fe^ii^	96.6 (4)	N2^iv^—Fe—Fe^i^	46.02 (18)
N1—Fe—Fe^ii^	83.4 (4)	N2^vii^—Fe—N2^v^	92.7 (5)
N1^iii^—Fe—Fe^i^	83.4 (4)	N2^vi^—Fe—N2^v^	87.3 (5)
N1^iii^—Fe—N1	180.0	N2^v^—Fe—N2^iv^	180.0 (7)
N1—Fe—N2^vii^	81.3 (4)	N2^vii^—Fe—N2^iv^	87.3 (5)
N1^iii^—Fe—N2^vii^	98.7 (4)	N2^vi^—Fe—N2^iv^	92.7 (5)
N1^iii^—Fe—N2^vi^	81.3 (4)	N2^vi^—Fe—N2^vii^	180.0
N1—Fe—N2^vi^	98.7 (4)	N1^viii^—N1—Fe	118.9 (9)
N1—Fe—N2^iv^	90.5 (5)	N1^viii^—N1—N2	109.6 (9)
N1—Fe—N2^v^	89.5 (5)	N2—N1—Fe	129.4 (9)
N1^iii^—Fe—N2^v^	90.5 (5)	Fe^ix^—N2—Fe^x^	87.3 (5)
N1^iii^—Fe—N2^iv^	89.5 (5)	N1—N2—Fe^x^	110.9 (12)
N2^vi^—Fe—Fe^i^	46.7 (5)	N1—N2—Fe^ix^	126.9 (5)
N2^vi^—Fe—Fe^ii^	133.3 (5)	N1—N2—N2^xi^	107.6 (12)
N2^vii^—Fe—Fe^i^	133.3 (5)	N2^xi^—N2—Fe^x^	113.1 (6)
N2^v^—Fe—Fe^ii^	46.02 (18)	N2^xi^—N2—Fe^ix^	109.9 (9)
N2^v^—Fe—Fe^i^	133.98 (18)		

Symmetry codes: (i) *x*+1, *y*, *z*; (ii) *x*−1, *y*, *z*; (iii) −*x*+1, −*y*, −*z*; (iv) *x*+1, *y*, *z*+1; (v) −*x*, −*y*, −*z*−1; (vi) −*x*+1, −*y*, −*z*−1; (vii) *x*, *y*, *z*+1; (viii) −*x*, −*y*−1, −*z*−1; (ix) *x*, *y*, *z*−1; (x) *x*−1, *y*, *z*−1; (xi) −*x*, −*y*−1, −*z*−2.
